# Urinary tritium concentrations and associated environmental factors among residents living near a nuclear power plant in South Korea: a community-based biomonitoring study

**DOI:** 10.1093/jrr/rrag068

**Published:** 2026-08-21

**Authors:** Minyu Qin, Keon Wook Kang, Hyun Chul Lee, Yong Hoon Jeong, Seokyoung Ahn, Insuk Song, Sang Jun Han, Seok-Ju Yoo, Ji-Hyuk Park, JaeYun Lee, JaeSeon Ryu, HyunChul Park, Kwan Lee

**Affiliations:** Department of Preventive Medicine, College of Medicine, Dongguk University, 123, Dongdae-ro, Gyeongju 38066, Republic of Korea; Department of Nuclear Medicine, Seoul National University Hospital, 101, Daehak-ro, Jongno-gu, Seoul 03080, Republic of Korea; Cancer Research Institute, Seoul National University College of Medicine, 202-1 Changgyeonggung-ro, Jongno-gu, Seoul 03080, Republic of Korea; Nuclear System Division, School of Mechanical Engineering, Pusan National University, 2, Busandaehak-ro 63beon-gil, Geumjeong-gu, Busan 46241, Republic of Korea; Department of Nuclear and Quantum Engineering, Korea Advanced Institute of Science and Technology, 291 Daehak-ro, Daejeon 34141, Republic of Korea; School of Mechanical Engineering, Pusan National University, 2, Busandaehak-ro 63beon-gil, Geumjeong-gu, Busan 46241, Republic of Korea; Wolsong Nuclear Facility Environment and Safety Monitoring Committee, 19-6, Neunggoryeondang-gil, Munmudaewang-myeon, Gyeongju 38119, Republic of Korea; RMTEC Co., Ltd, 25 Hanamsandancheonbyeonjwa-ro, Gwangsan-gu, Gwangju 62215, Republic of Korea; Department of Preventive Medicine, College of Medicine, Dongguk University, 123, Dongdae-ro, Gyeongju 38066, Republic of Korea; Department of Preventive Medicine, College of Medicine, Dongguk University, 123, Dongdae-ro, Gyeongju 38066, Republic of Korea; Department of Preventive Medicine, College of Medicine, Dongguk University, 123, Dongdae-ro, Gyeongju 38066, Republic of Korea; Department of Preventive Medicine, College of Medicine, Dongguk University, 123, Dongdae-ro, Gyeongju 38066, Republic of Korea; Department of Preventive Medicine, College of Medicine, Dongguk University, 123, Dongdae-ro, Gyeongju 38066, Republic of Korea; Department of Preventive Medicine, College of Medicine, Dongguk University, 123, Dongdae-ro, Gyeongju 38066, Republic of Korea

**Keywords:** tritium, nuclear power plant, drinking water, environmental exposure, biological monitoring

## Abstract

Abnormally high tritium levels were identified at the Wolsong nuclear power plant (NPP) in 2019, prompting growing concern among nearby residents and public health authorities. The study aimed to investigate tritium internal exposure concentrations among residents and to identify potential internal exposure pathways. A total of 359 residents living within 15 km of the NPP with no occupational history at the facility were enrolled in this cross-sectional study in 2021. Participants were categorized into three distance-based zones. Tritium concentrations were measured in urine samples and local water resources. Statistical analyses were conducted using appropriate parametric tests and linear regression based on log-transformed data. The results showed that residents within 10 km of the NPP exhibited significantly higher urinary tritium levels (mean ± SD: 3.28 ± 2.28 Bq/L; 95% CI: 2.87–3.69; *P* < 0.05) compared with those living further away. Tap water within 5 km contained the highest tritium levels (6.6 ± 1.6 Bq/L), whereas groundwater in the 5–10 km zone showed elevated levels (2.0 ± 1.9 Bq/L). Participants who had visited the NPP in the previous 2 years presented higher urinary tritium levels than those who had not (*P* < 0.01). Overall, this study showed that urinary tritium concentrations varied according to residential distance and water resource. All measured urinary tritium levels were far below international safety limits. These findings establish essential baseline data for future surveillance and risk communication on nuclear-related public health issues.

## INTRODUCTION

The Wolsong nuclear power plant (NPP) in South Korea operates as a heavy-water reactor, using heavy water as both a moderator and a coolant. The heavy-water reactor is known to produce higher levels of radioactive tritium compared to other reactor types [1, 2]. The health risks associated with radiation exposure have always been a concern in public health discussions. In April 2019, an abnormally elevated tritium concentration of 713.000 Bq/L was detected in the turbine gallery manhole of Wolsong Unit 3 [3]. The discovery provoked considerable public anxiety regarding a potential radioactive leak. The Korean Nuclear Safety and Security Commission (NSSC) later conducted studies and determined that the elevated concentration was caused by subterranean tritium migration rather than an active leak [3]. Nevertheless, public concern over potential health impacts persisted.

Tritium emits low-energy beta radiation that cannot penetrate the cell nucleus but may contribute to internal exposure when inhaled, ingested or absorbed [4]. Residents living near nuclear facilities may be exposed internally to radionuclides, particularly tritium, through different pathways, including inhalation, ingestion and dermal absorption [5]. Environmental research has also shown that tritium can spread through air, water and human biological samples [2], raising the possibility of internal exposure in surrounding communities. However, the effects of tritium exposure on populations are still controversial, especially at low levels. Even though previous epidemiological studies have indicated a higher risk of thyroid cancer among residents living close to NPPs [6, 7]. It is challenging to determine the exact contribution of tritium exposure because these studies have not separated the effects of tritium from other radionuclides. The health implications of low-dose tritium remain difficult to quantify, as environmental concentrations typically lie far below regulatory thresholds and even below background natural sources (13 mBq/L) [8]. Additionally, the health effects of low-level tritium exposure have been demonstrated only in animal studies. The researchers found sub-cellular effects, including genotoxicity, activation of DNA repair pathways, and changes in antioxidant and immune function in aquatic organisms [9, 10].

This study aimed to estimate biological tritium exposure among residents living near the Wolsong heavy-water reactor and to explore potential environmental pathways associated with internal exposure. Additionally, this study aimed to provide evidence supporting public risk communication at low-level exposure around nuclear facilities.

## METHOD

### Data collection

Our study recruited residents from three regions surrounding the Wolsong NPP between August 30 and 15 September 2021. The study focused on a quota-based community sampling design across villages located within a 15 km radius of the Wolsong NPP, specifically in three geographical regions: Gampo-eup, Mummudaewang-myeon and Yangnam-myeon, referred to as A-area, B-area and C-area, respectively. A total of 38 villages were included, categorized into three distance ranges: within 5 km, 5–10 km, and 10–15 km from the plant, as shown in Fig. 1. The distance-based classification was established based on the decline of radionuclide concentrations in line with previous epidemiological and environmental studies around NPP [11, 12]. Similar methodologies were utilized in these studies to evaluate potential radiation exposure and health-related outcomes in South Korea. Purposive quota sampling was employed to ensure proportional representation based on the population of each area. We selected 120 residents from each region, balancing by gender and age.

**Fig. 1:**
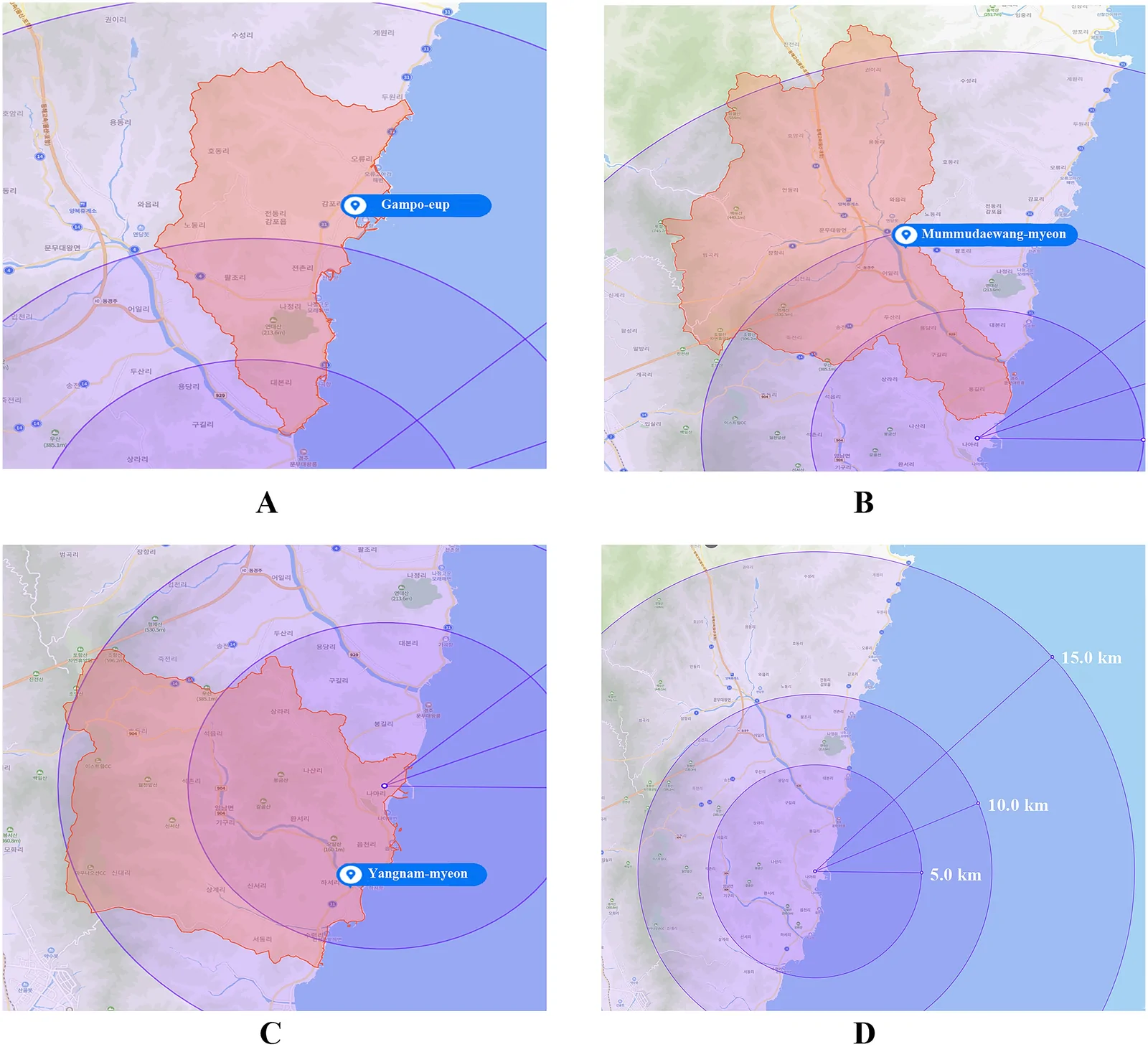
Geographical location of the Wolsong nuclear power plant (NPP) and participating villages. Panels A–C represent the three geographical study regions surrounding the Wolsong NPP. Panel D presents an overview of all study villages included in the study. A: 1 village within 5 km, 3 within 10 km, and 5 within 15 km of the Wolsong NPP; B: 3 villages within 5 km, 5 within 10 km, and 7 within 15 km; C: 11 villages within 5 km and 4 within 10 km; D: overview of all villages located within 15 km of the Wolsong NPP.

The sample size was estimated using the following function and based on an expected tritium detection rate of 47.9%, derived from preliminary regional monitoring data (expected detection rates of 16 150 residents among three regions were 31.3%, 45.6% and 66.7%, respectively). The survey was designed with a sampling error of 5–10% at a 95% confidence interval. The estimated required sample size ranged from 96 to 376 participants. Consequently, given the need to ensure balanced representation across three geographic, sex and age groups, 360 residents were recruited for this study. A total of 359 participants were ultimately included in the final analysis, with one participant excluded due to occupational exposure.


$$ N=\frac{Z^2\times p\times \left(1-p\right)}{E^2} $$

The Z represents the Z value corresponding to the desired confidence level (e.g. 1.96 for 95% confidence level). In addition, P denotes the proportion of respondents selecting a particular choice, expressed as a decimal. Furthermore, E signifies the margin of error, or confidence interval, also expressed as a decimal.

The inclusion criteria were as follows: residents who had lived in A, B and C areas for at least 1 year. Although the study eligibility criteria included individuals aged 14 years and older, no participants younger than 18 years of age were enrolled in the final study population. Participants were non-NPP workers; individuals with prior experience working at the nuclear facility were excluded to eliminate potential bias related to occupational exposure. Face-to-face questionnaires were administered to study participants, covering demographic characteristics (sex, date of birth, address, occupation, residency years, daily activities & time) and dietary habits (serving size, serving times, food forms, meat consumption, fried food consumption, stir-fried rice consumption, type of drinking water used—groundwater, bottled drinking water or tap water—and frequency of local agricultural and fish products intake). The study protocol is outlined in Fig. 2.

**Fig. 2:**
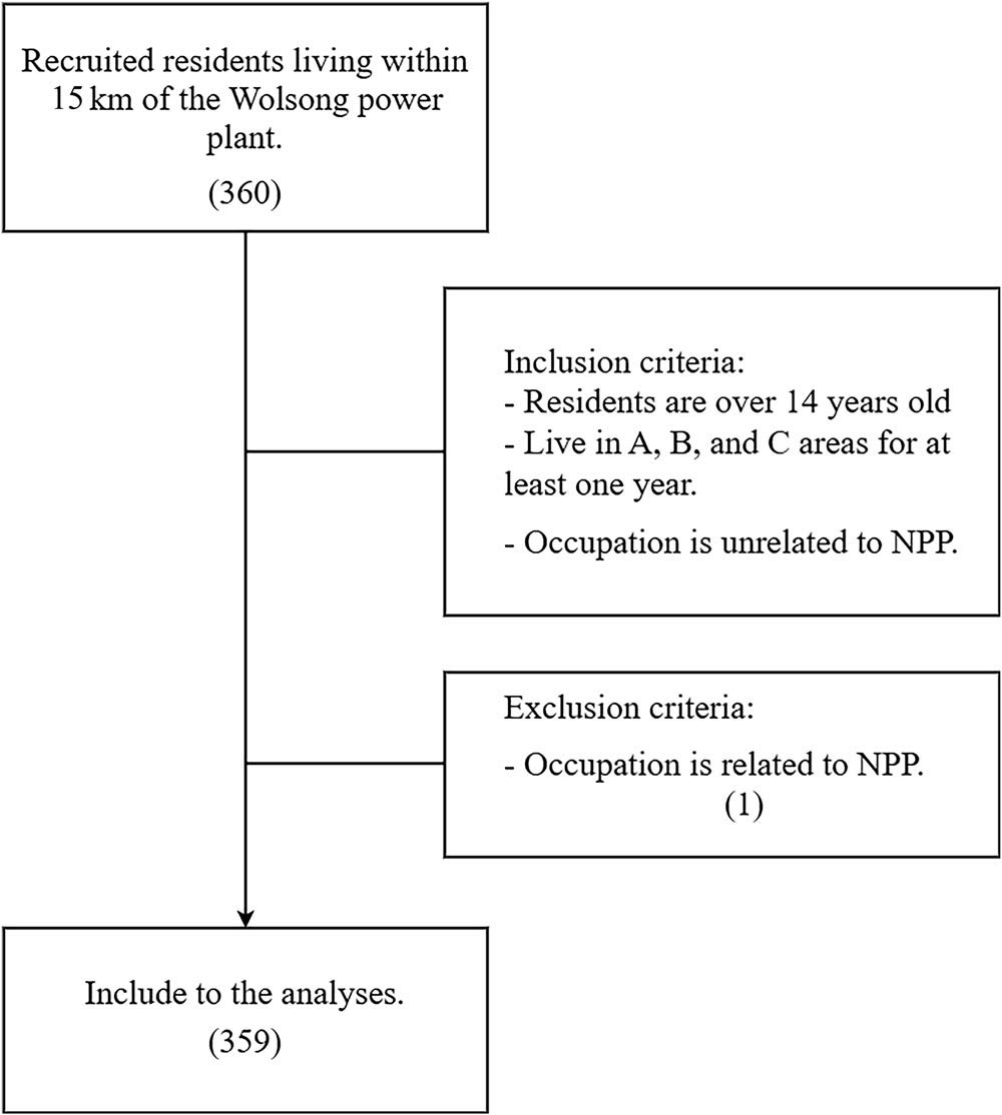
Protocol of data collection. A total of 359 participants were included in the study, of whom 360 residents completed the survey, and one individual reported having worked at the Wolsong nuclear power station (2021).

### Measurement of tritium

Urine samples exceeding 50 ml were collected from participants for tritium measurement. To prevent spoilage, the specimens were refrigerated and transported to the medical center within 24 h. Before analysis, the samples underwent heat evaporation followed by chemical treatment using KMnO_4_ and Na_2_O_2_. A conservative interpretation threshold for tritium detection was established at 2 Bq/L. However, the actual analytical minimum detectable activity (MDA) of the liquid scintillation counting system was typically lower due to different measurement conditions, such as sample volume, counting time and detector efficiency. A total of 163 samples (45.4%) were assigned the sample-specific MDA value. Urinary tritium concentration was used as a biomarker of internal tritium exposure because tritiated water (HTO) rapidly equilibrates with body water and is primarily eliminated through urine. Therefore, urinary tritium levels are widely used for biological monitoring of internal tritium exposure in environmental and occupational studies [5].

### Statistical analysis

Annual effective doses were estimated using the International Commission on Radiological Protection (ICRP) dose coefficient for tritiated water (2.0 × 10^−8^ mSv/Bq), assuming equilibrium between tritium intake and excretion and an annual drinking water consumption of 730 L per person. Urinary tritium concentrations are presented as original values (Bq/L) for descriptive purposes. Geometric mean (G.M.) ± Geometric Standard Deviation. (G.S.D.) were used to summarize internal tritium concentrations. Because of the right-skewed distribution, a national logarithmic transformation was performed for inferential statistical analyses. Log-transformed urinary tritium concentrations were used for group comparisons and regression analyses. The distribution of transformed data was evaluated using the Shapiro–Wilk test and normal Q-Q plots (shown in Fig. S1). Student’s *t-*test or one-way ANOVA was applied to group comparisons. Multiple linear regression based on the ordinary least squares method was performed using transformed data as the dependent variable. The model assumptions were evaluated using standardized residual histograms, normal P–P plots, residual-versus-predicted plots and Cook’s distance (Fig. S1).

### Ethics approval and consent to participate

Informed consent was obtained from all the participants for their participation in this study. Prior explanation of the study was provided, and consent was obtained from the participants. The study was conducted in accordance with the established procedures of the institutional review board and the 1964 Helsinki Declaration. All consent forms were received in writing and were stored in a file. The Institutional Review Board approved the study protocol (IRB No. 110757-202107-HR-01).

## RESULTS

The demographic characteristics of the study participants by region are presented in Fig. 3. Among the participants, 190 were men (52.8%) and 170 were women. In terms of age, 221 participants (61.6%) were in their 60s, 83 (23.1%) were in their 50s, and 35 (9.7%) were in their 40s. The average tritium concentration among participants was 3.72 Bq/L, with a geometric mean of 2.55 Bq/L. The 90th and 95th percentiles, were 7.08 Bq/L and 8.66 Bq/L.

**Fig. 3:**
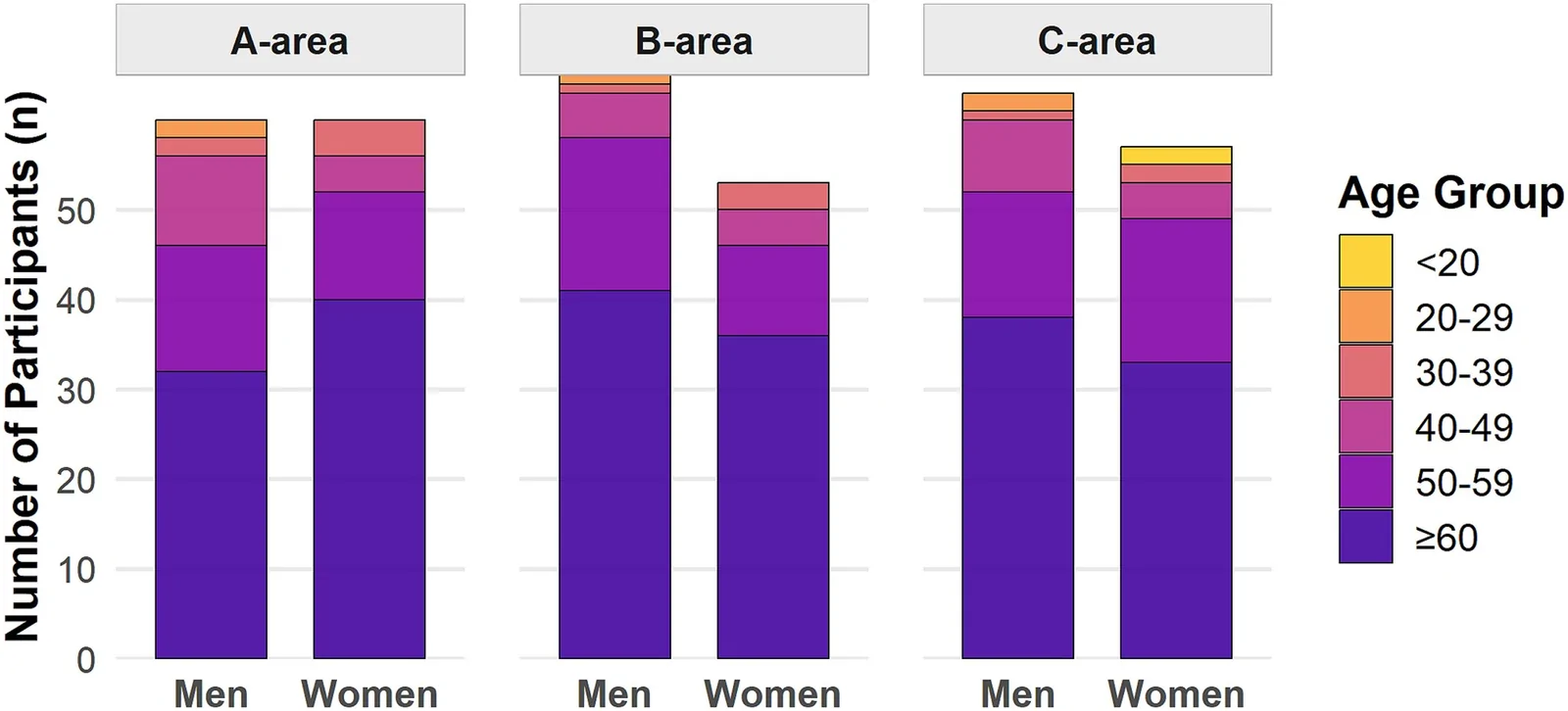
Demographic distribution of participants by region (*n* = 359).

We analyzed characteristics for urinary tritium concentration from four aspects – geographical, gender, age and residential duration, as shown in Table 1. Region differences were significantly observed, with participants from the C-area exhibiting the highest tritium levels (3.28 Bq/L), significantly higher than those from the B-area (2.35 Bq/L) and A-area (2.08 Bq/L) (*P* < 0.001). Although men showed slightly higher tritium levels compared to women, the difference was not statistically significant. Furthermore, no significant age-related, and residential duration-related differences were detected. The general dosage of urinary concentration is 2.53 ± 2.09 Bq/L.

**Table 1:** Tritium concentrations in subjects from different regions, genders and age groups (*n* = 359) (Bq/L)

Variables	n	Mean ± S.D.	Median	Minimum	Maximum	G.M. ± G.S.D.
Regions^**a**^						
A-area	120	2.51 ± 1.75	1.77	1.05	13.10	2.08 ± 1.81
B-area	119	3.07 ± 2.44	1.38	1.16	11.90	2.35 ± 2.03
C-area	120	4.61 ± 4.57	3.7	1.14	39.30	3.28 ± 2.28^*^
Sex^**b**^						
Men	189	3.67 ± 3.69	2.84	1.05	39.30	2.71 ± 2.12
Women	170	3.10 ± 2.73	1.43	1.05	16.30	2.34 ± 2.05
Age^**a**^						
<20	2	5.94 ± 4.85	5.94	2.51	9.37	4.85 ± 2.54
20–29	5	3.88 ± 2.49	3.75	1.3	7.92	3.29 ± 1.93
30–39	13	3.30 ± 3.41	2.34	1.21	13.70	2.41 ± 2.12
40–49	35	3.56 ± 3.01	2.37	1.09	12.10	2.59 ± 2.21
50–59	83	3.26 ± 2.59	2.59	1.05	13.10	2.48 ± 2.08
>60	221	3.40 ± 3.55	2.57	1.05	39.30	2.51 ± 2.09
Residential duration (years)^**a**^						
<10	57	3.61 ± 5.47	1.34	1.09	39.30	2.33 ± 2.24
10–19	63	3.04 ± 2.36	2.51	1.14	13.70	2.39 ± 1.98
≥20	238	3.44 ± 2.73	2.79	1.05	16.30	2.61 ± 2.09
Total	359	3.40 ± 3.27	2.57	1.05	39.30	2.51 ± 2.09

Urinary tritium concentrations are presented as geometric means (G.M.) based on the original values (Bq/L). *P*-values were calculated using log-transformed urinary tritium concentrations.

G.M.: geometric mean; G.S.D.: Geometric Standard Deviation.

^*^ *P* < 0.001.

^a^One-way ANOVA.

^b^Student’s *t-*test.

The association between distance from the NPP and urinary tritium levels was investigated by linear regression (Fig. 4). As illustrated, the model provided a low but statistically significant coefficient of determination (R^2^ = 0.29), suggesting that residential distance accounts for ~30% of the variation in internal tritium exposure. After stratifying by region, the C-area showed the highest correlation (R^2^ = 0.47), followed by the B-area and A-area (Fig. 4A–C). This geographical variability suggests that factors other than distance may contribute to internal tritium exposure. Potential influences reported in previous studies include hydrology, atmospheric dispersion and local dietary behaviors. However, these variables were not available in the present dataset and therefore could not be evaluated in the regression model. Further exposure assessment models must incorporate behavioral, nutritional and environmental data because of the significant unexplained variance (>70%), which emphasizes the multifactorial nature of individual tritium exposure.

**Fig. 4:**
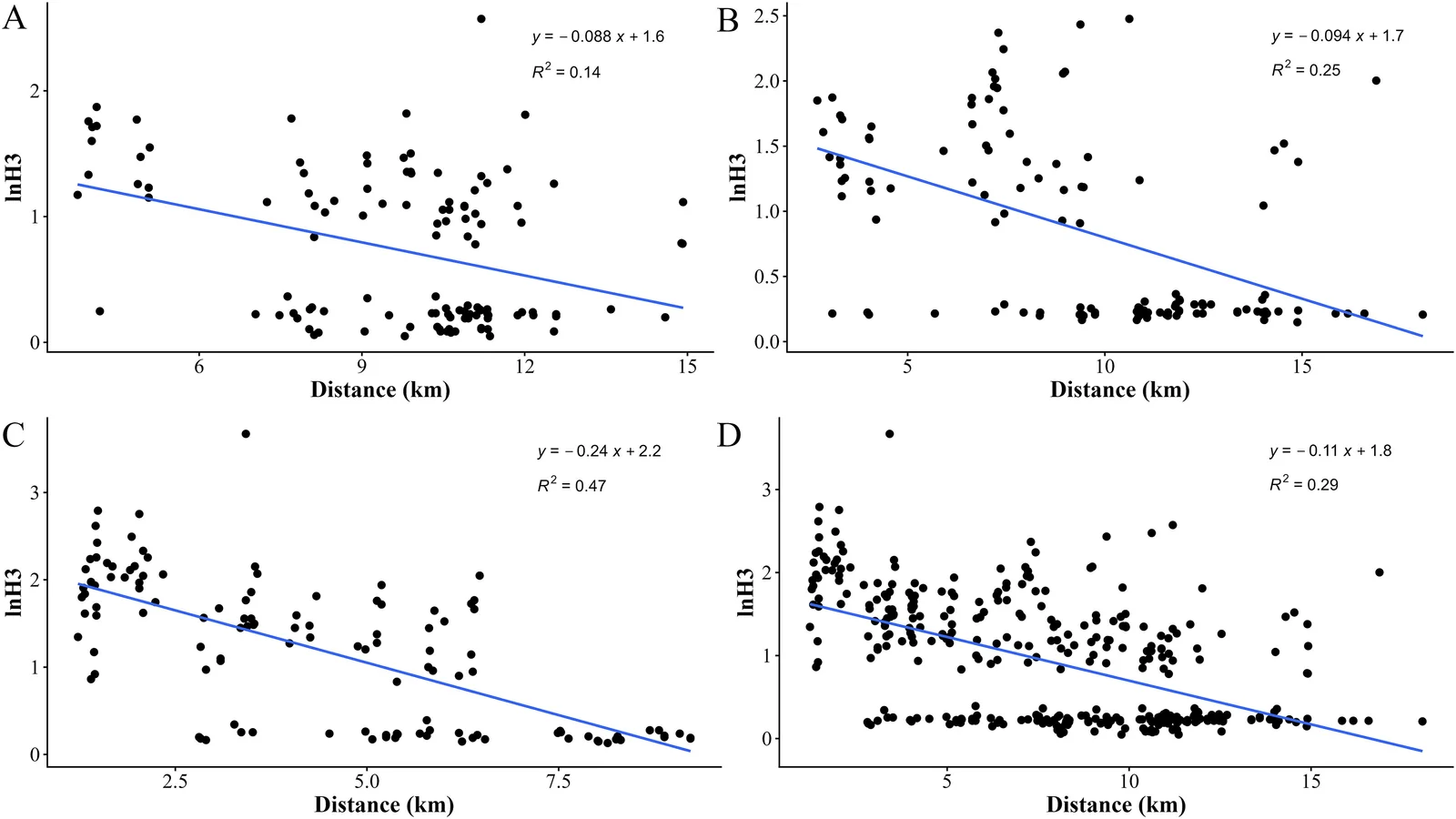
Linear regression analysis of log-transformed urinary tritium concentrations according to residential distance. A: Correlation between log-transformed tritium concentration and distance from A-area (*n* = 120); B: Correlation between log-transformed tritium concentration and distance from B-area (*n* = 119); C: Correlation between log-transformed tritium concentration and distance from C-area (*n* = 120); D: Correlation between log-transformed tritium concentration and distance from 3 regions (*n* = 359). lnH3: Log-transformed tritium.

Tritium concentration in drinking water sources was also evaluated to assess its impact on daily usage by the population (shown in Table 2). Overall, tritium levels demonstrated significant variations between tap water and groundwater. Our notable findings observed significant spatial variation within the C-area. Tritium levels in tap water within 5 km of the NPP were significantly higher (G.M. = 6.6 Bq/L) than in the groundwater. In the 5–10 km range, groundwater showed higher tritium concentrations (G.M. = 2.0 Bq/L) compared to tap water (1.2 Bq/L).

**Table 2:** Tritium concentration in different water resources (*n* = 359) (Bq/L)

Variables		n	Tritium concentration (G.M. ± G.S.D.)
Water resources	Total regions		
	Tap water	129	2.59 ± 2.16^*^
	Groundwater	230	2.49 ± 2.06
	C-area(<5 km)		
	Tap water	26	6.6 ± 1.6^*^
	Groundwater	41	4.3 ± 2.2
	C-area(5≤,<10 km)		
	Tap water	6	1.2 ± 1.0
	Groundwater	47	2.0 ± 1.9^**^

Urinary tritium concentrations are presented as geometric means (G.M.) based on the original values (Bq/L). *P*-values were calculated using log-transformed urinary tritium concentrations.

Statistical analyses used Student’s *t-*test; G.M.: Geometric Mean; G.S.D.: Geometric Standard Deviation.

^*^ *P* < 0.05.

^**^ *P* < 0.001.

Additionally, a history of visiting the NPP within the past 2 years was associated with significantly higher urinary tritium levels (G.M. = 5.28 Bq/L) compared to those with no visits (2.47 Bq/L), as shown in Table 3.

**Table 3:** Correlation of tritium concentration and visits in the last 2 years (*n* = 359) (Bq/L)

Assess history	n	Tritium concentration (G.M. ± G.S.D.)
Yes	10	5.28 ± 1.9
No	349	2.47 ± 2.08

Urinary tritium concentrations are presented as geometric means (G.M.) based on the original values (Bq/L). *P*-values were calculated using log-transformed urinary tritium concentrations.

*P* < 0.05 by Student’s *t-*test.

G.M.: geometric mean; G.S.D.: Geometric Standard Deviation.

Finally, we examined whether consumption of locally produced agricultural products influenced urinary tritium levels (Table 4). Most participants reported consuming local produce ‘Almost entirely’ (*n* = 99) or ‘More than 50%’ of the time (*n* = 177). However, no significant differences in tritium concentrations were observed across different consumption levels, suggesting that dietary intake of local agricultural products did not substantially contribute to internal tritium exposure.

**Table 4:** Tritium concentrations in agricultural products (*n* = 359) (Bq/L)

Intake of local agricultural products	n	Tritium concentration (G.M. ± G.S.D.)
Almost entirely	99	2.98 ± 2.03
More than 50%	177	2.17 ± 2.03
Less than 50%	55	2.83 ± 2.20
Basically no	28	2.93 ± 2.16
Summary	359	2.51 ± 2.09

Urinary tritium concentrations are presented as geometric means (G.M.) based on the original values (Bq/L). *P*-values were calculated using log-transformed urinary tritium concentrations. G.M.: geometric mean; G.S.D.: Geometric Standard Deviation.

*P* < 0.05 by one-way ANOVA.

The estimated annual effective doses derived from detected internal tritium concentrations were presented in Table 5. The overall geometric mean annual effective dose was 0.000037 mSv/year. The maximum geometric mean annual effective dose in the C-area reached 0.000574 mSv/year, corresponding to the highest observed urinary tritium concentration (39.3 Bq/L).

**Table 5:** Estimated annual effective dose derived from urinary tritium concentrations among study regions (*n* = 359) (unit: mSv/year)

Region	Mean	Median	Minimum	Maximum	G.M.
Overall	0.000050	0.000038	0.000015	0.000574	0.000037
A-area	0.000037	0.000026	0.000015	0.000191	0.000030
B-area	0.000045	0.000020	0.000017	0.000174	0.000034
C-area	0.000067	0.000054	0.000017	0.000574	0.000048

Estimated annual effective dose was calculated using urinary tritium concentration, an annual drinking-water intake of 730 L/year (2 L/day), and the ICRP dose coefficient for tritiated water (2.0 × 10^−8^ mSv/Bq). G.M.: geometric mean.

## DISCUSSION

Urinary tritium concentrations were used to assess the internal exposure among residents living near Wolsong NPP, a heavy-water reactor. The measured levels were generally low, with a mean of 2.53 ± 2.09 Bq/L, and a maximum of 3.28 ± 2.28 Bq/L within the study population. The significant spatial gradient and water source variation were observed. In contrast, the demographic factors such as age, sex and residence duration were insignificantly associated with tritium level, revealing that environmental factors are the primary determinants of internal exposure. These findings provide a quantitative basis for subsequent dose estimation.

Internal radiation dose was estimated using the ICRP dose coefficient (2.0 × 10^−8^ mSv/Bq) and urine tritium concentrations, assuming equilibrium between daily intake and excretion. The tritium concentration among residents within 15 km of the NPP (2.55 Bq/L) is comparable to the previous studies, one of which measured residents’ tritium concentrations within 22 km of heavy-water reaction in China (1.26–6.73 Bq/L) [5], and another one of the general South Korean public (2.8 ± 1.4 Bq/L) [13]. The maximum observed urinary tritium concentration corresponded to an estimated annual effective dose in our study, which is higher than the above. The calculated annual dose in the C-area with the highest mean concentration was 0.000048 mSv, while the average for all participants was ~0.000037 mSv. These values are several orders of magnitude below the natural background radiation level in South Korea [14] and significantly lower than the public exposure limit of 1 mSv/year recommended by the ICRP [15]. These findings provide quantitative evidence for evaluating low-level environmental radiation exposure, despite the very low level of tritium detected. The estimated doses remain negligible in terms of radiological health risk, even under conservative assumptions.

Urinary tritium concentrations demonstrated a distinct spatial variation throughout the study locations. Notably, the C-area in the southwest of the plant, with the majority living within 5 km, showed the greatest concentrations. Nevertheless, the northwestern and northern regions, where most of them live far away from 10 km, had a lower level, suggesting a diverse spatial distribution. Such spatial patterns are consistent with the previous studies showing that proximity to emission sources is a major factor in determining internal exposure [5, 16]. Tritium concentrations in environmental media decrease with increasing distance from the nuclear sites. In addition to that, groundwater and soil systems were found to show comparable spatial variability in the previous research [17, 18]. However, our findings showed a much greater urinary tritium concentration among tap water users within 5 km than among groundwater users. In contrast, a completely different outcome was seen beyond 5 km. The variation implies that traditional distance-based gradients might not be adequate to explain individual exposure levels due to ignoring variations in environmental transport and absorption mechanisms. Previous studies on light-water reactor (LWR) surveillance reports showed generally lower tritium concentrations than those in heavy-water reactors [19]. The tritium concentrations. For example, monitoring studies in Taiwan and France showed that tritium concentrations in drinking water near LWR facilities were generally close to regional background levels and were not consistently associated with specific drinking-water sources. In contrast, heavy-water reactors produce substantially larger amounts of tritium through neutron activation of deuterium in moderator and coolant systems. Recent analyses of Korean NPPs demonstrated that tritium releases from pressurized heavy water reactors were approximately nine times higher than those from pressurized water reactors [20]. Therefore, localized hydrological influences on tritium distribution may be more readily detectable around heavy-water reactor sites such as Wolsong. Additionally, variations in water resources may be partially reflected by the characteristics of the local water supply system, such as geographic and infrastructural factors, rather than the direct effect of the water source alone. In the study areas, residents farther from the NPP are more likely to rely on groundwater because centralized tap water services are less available, while the municipal water treatment facility is located ˃15 km from the plant. Nevertheless, the available evidence remains insufficient to determine whether the higher urinary tritium concentrations observed among tap-water users represent a characteristic feature of heavy-water reactor environments, and additional comparative studies across reactor types are warranted. Tritium incorporation into surface water systems may be facilitated by localized hydrological processes and soil-water interactions [21]. Surface water systems may respond more rapidly to atmospheric deposition and precipitation events than groundwater systems, which are generally buffered by longer residence times and subsurface transport processes [22]. Routine environmental radiation monitoring programs are regularly conducted around South Korean NPPs. The monitoring results include tritium measurements in precipitation, surface water, drinking water and groundwater, which support the description of regional hydrological systems and support the plausibility of environmental transport pathways involving atmospheric deposition and water circulation. However, the precipitation influence could not be directly evaluated, because concurrent precipitation monitoring results were not included in the study design.

The observed directional differences suggest that environmental or geographic factors other than residential distance may contribute to spatial variability in tritium distribution. Previous studies have reported that atmospheric dispersion processes, including prevailing wind patterns, may influence environmental tritium transport. However, meteorological variables were not collected in the present study and therefore could not be incorporated into the regression analysis. Consequently, the potential contribution of atmospheric factors remains speculative and warrants further investigation in future studies. However, meteorological data were not included in this analysis. More research is therefore needed to determine the mechanisms driving the observed regional heterogeneity. Last but not least, the sampling period (August–September) may also have an impact on the regional patterns, which indicate the seasonality. Previous research has found seasonal variation in atmospheric tritium concentrations [23, 24], which is frequently connected with changes in humidity, precipitation and atmospheric mixing conditions. The current investigation was carried out in late summer (August–September), which is characterized by relatively high humidity and rapid hydrological cycling, which may accelerate tritium transfer from the atmosphere to surface water. Although these external processes may help to explain the observed patterns of internal exposure, it is important to emphasize that meteorological and temporal data were not explicitly incorporated into the analysis. As a result, longitudinal and process-based studies are needed to better understand the relative contributions of emission characteristics and seasonal factors to tritium distribution.

Regarding tritium is released in both gaseous and liquid forms during NPP, mostly as HTO [19, 25]. Gaseous emissions are emitted into the atmosphere as water vapor or other hydrogen-containing molecules, while liquid effluents are dumped into nearby water systems. According to publicly accessible monitoring data, gaseous tritium emissions from the study location have historically outpaced liquid discharges [26]. According to Korea Nuclear Safety’s disclosure, the emission of tritiated water vapor from Wolsong NPP was more than three times higher than liquid emissions in 2019 and prior years, with an average emission of 19.33 TBq compared to 4.90 TBq for liquid emissions. From 2020 to June 2024, gaseous tritium emissions were controlled (Average emission = 6.81 TBq), while liquid emissions increased (Average emission = 9.95 TBq) [26]. Concentrations of tritium in water sources were found to be below the World Health Organization threshold of 10 000 Bq/L [27]. Emissions of tritium around the Wolsong NPP and tritium urinary concentration in residents seemed to remain below established safety thresholds.

Beyond its environmental significance, this study also provides information relevant to risk communication in communities surrounding heavy-water reactor facilities. Because tritium can be detected at extremely low concentrations, public concern is often driven by the presence of measurable radioactivity rather than by the actual magnitude of exposure. Therefore, risk communication strategies should emphasize quantitative comparisons between observed exposure levels, natural background radiation and regulatory dose limits. Although spatial variations in urinary tritium concentrations were observed in this study, the measured concentrations and estimated annual effective doses remained extremely low and far below internationally recommended public exposure limits. Therefore, communicating not only the presence of detectable tritium but also the magnitude of exposure and its associated health significance is essential. Regular environmental and biological monitoring, coupled with transparent reporting of results, may help reduce unnecessary anxiety, improve public understanding of tritium exposure pathways, and support evidence-based decision-making among communities residing near NPPs.

Some limitations should be considered in this study. Firstly, this cross-sectional study relied on a single urinary tritium measurement and lacked long-term biomonitoring data. Consequently, temporal variations in internal tritium exposure and cumulative dose could not be evaluated. Secondly, the lack of participants under 40 years old affected the adaptability of this study. Thirdly, the variability in environmental factors, such as regional differences in water quality and agricultural practices, could influence the results. Additionally, the complexity of accurately measuring low-level internal exposure to tritium poses challenges in drawing definitive conclusions about its long-term health effects, particularly concerning cancer and genetic diseases. Furthermore, environmental and meteorological parameters, including wind direction, wind speed and precipitation-related tritium monitoring data, were not available during the study period. Therefore, the potential influence on the spatial distribution of urinary tritium concentrations could not be directly assessed.

## CONCLUSION

Tritium concentrations in residents near the Wolsong NPP—including non-occupationally exposed individuals—were consistently negligible, with monitored water sources remaining well below regulatory limits. These results suggest that the associated doses are negligible, posing no discernible health risk. To promote the residents’ awareness of nuclear radiation and its true health effects at different doses, we should engage in active communication with the communities and residents, offer scientific education about nuclear radiation, and frequently share the findings of peripheral radiation dose monitoring.

## Supplementary Material

Supplementary_material_rrag068 Distribution of log-transformed urinary tritium concentrations and diagnostic plots for the multiple linear regression model. (A) Normal Q-Q plot of log-transformed urinary tritium concentrations. (B) Histogram of standardized residuals. (C) Normal P-P plot of standardized residuals. (D) Scatterplot of standardized residuals versus standardized predicted values.

## Data Availability

All data generated or analyzed during this study are included in this article. Further inquiries can be directed to the corresponding author.

## PRESENTATION AT A CONFERENCE

This work has not been presented at any conference.
